# Potentially Toxic Element Pollution Levels and Risk Assessment of Soils and Sediments in the Upstream River, Miyun Reservoir, China

**DOI:** 10.3390/ijerph15112364

**Published:** 2018-10-25

**Authors:** Libo Pan, Guangling Fang, Yue Wang, Lei Wang, Benying Su, Dan Li, Bao Xiang

**Affiliations:** 1 Chinese Research Academy of Environmental Sciences, Agricultural Environmental Research Center, Beijing 100012, China; panlb@craes.org.cn (L.P.); fanggl@craes.org.cn (G.F.); wangyue@craes.org.cn (Y.W.); wanglei@craes.org.cn (L.W.); shuying5211@126.com (B.S.); 2 Chinese Research Academy of Environmental Sciences, State Key Laboratory of Environmental Criteria and Risk Assessment, Beijing 100012, China; lidan@craes.org.cn

**Keywords:** potentially toxic element, pollution level, soil, sediment, risk assessment

## Abstract

This study focused on the Chao River and Baimaguan River located upstream of the Miyun Reservoir in Miyun District (Beijing, China). Soil and sediment samples were collected from the river and drainage basin. Total nitrogen, total phosphorus, and six potentially toxic elements including cadmium, zinc, lead, chromium, arsenic, and copper, were analyzed in terms of concentration, potential ecological risk, and human health risk. The average concentrations of the six potentially toxic elements were all below the soil environmental quality standards for China. Cadmium was the most serious pollutant in both soils and sediments, at 2.58 and 3.40 times its background values. The contents of Cd and Pb were very closely related (*p* < 0.01) to total nitrogen concentrations in both soil and sediment samples. The potential ecological risks posed by Cd in the Chao and Baimaguan River soils were considerable and moderate, respectively. The historical iron ore mining and agricultural activity were identified as the primary sources of potentially toxic element pollution of soil and sediment in the Chao-Bai River in Miyun District. Human health risk assessment indicated that non-carcinogenic risks all fell below threshold values. The total carcinogenic risks due to Cr and As were within the acceptable range for both adults and children. This conclusion provides a scientific basis for the control of potentially toxic element pollution and environmental protection of the Miyun Reservoir in Beijing.

## 1. Introduction

Potentially toxic elements are naturally occurring, ubiquitous substances in the human environment, which typically originate from the weathering of parent materials. Nevertheless, due to a variety of human activities, including mineral resources development, metal processing and smelting, industrial emissions, application of fertilizers and pesticides, sewage irrigation, and atmospheric transportation [1,2], potentially toxic elements have substantially accumulated in the global environment in recent years, particularly in soil and sediment environments. According to the national communique of soil pollution survey by the Ministry of Environmental Protection of China and the Ministry of Land and Resources in 2014 [3], soil has been contaminated heavily in some regions, and the quality of cultivated land soil is particularly concerning. Potentially toxic elements are transported in various forms through the exchange of substances among ecosystems, as they are highly mobile in air and water.

The transformation and migration patterns of potentially toxic elements in the aquatic environment are complex processes. Potentially toxic elements in the sediments are mainly from two sources, i.e., natural sources such as rock weathering and anthropogenic activities such as mining and agriculture. For example, Cd is considered to derive mainly from phosphorous fertilizers [4]. Zinc and Cd found in water are typically from mining activities or industrial effluents, as well as combustion of fossil fuels [5]. In addition, some studies have also indicated that fine sediments are carriers of potentially toxic elements in the surface land, flowing into the aquatic environment by surface runoff [5]. In the aquatic environment, potentially toxic elements can migrate with a carrier, and are eventually absorbed by fine mineral particles in sediments and soils, leading to potentially toxic element pollution in aquatic environments, which has the potential to cause serious aquatic ecosystem and human health impairments [6]. Elements such as metals dissolved in natural waters are easily absorbed by aquatic organisms and can rapidly bioaccumulate/biomagnify within the aquatic food web. Chronic potentially toxic element exposure in aquatic ecosystems may adversely affect the activity, growth, metabolism, and reproduction of aquatic organisms [7]. Major Chinese rivers including the Chang River, Huang River and Zhu River are contaminated with potentially toxic elements to various degrees; Cd and Hg are the most heavily polluting elements, and are categorized as having high and extremely high ecological risks, respectively [8]. In China, approximately 10 million ha of arable land has been polluted, and approximately 12 million tons of grain is contaminated every year due to potentially toxic elements in soils [4].

In recent years, review and research articles have provided assessments of various kinds of soil heavy metal pollution all over the world, especially in developing countries, including urban soil, agricultural soil and mining area soil, such as the mining areas in Shanxi [9,10,11] and other provinces in China [11], as well as agricultural areas in southeast and northwest China [12,13]. Such studies have helped to raise public awareness of soil contamination and to facilitate research for pollution control. On the national scale, Niu et al. [14] reviewed metal accumulation across Chinese main farmland soil, Cd was found to have the highest pollution index (PI) of 5.28; Chen et al. [2] identified the contamination characteristics of heavy metals in Chinese soils, the results revealed that cadimium and mercury were identified as the priority control metals, and indicated moderate contamination level. Yang et al. [15] assessed potentially toxic element pollution and associated risks of industrial and agricultural soils in China, cadmium, lead, and arsenic showed more serious effects, and those in southeast China were severer than those in northwest China. In addition to China, potentially toxic element pollution studies were also conducted in other countries, i.e., Vietnam, Brazil, Congo, Thailand, where potentially toxic elements showed various contamination levels, and coal combustion, industrial and domestic waste discharges, and agricultural activity are the main sources [16,17,18,19].

Potentially toxic elements, especially heavy metals, can enter food chains by enrichment, posing risks to the ecosystem and human health through long-term exposure, even at very low concentrations. For example, Pb is a non-essential element for humans, and excessive intake can damage the nervous, skeletal, circulatory, enzymatic, endocrine, and immune systems [20]. Chronic exposure to Cd can have adverse effects such as lung cancer, pulmonary adenocarcinomas, prostatic proliferative lesions, bone fractures, kidney dysfunction, and hypertension. Meanwhile, the chronic effects of As exposure include dermal lesions, peripheral neuropathy, skin cancer, and peripheral vascular disease [21]. Nowadays, A “war” to conquer soil pollution is occurring in China to improve soil quality, ensure the quality of agricultural products and protect the health of humans as well as animals. For example, a national action plan called “Soil Ten Chapter” has been implemented for the sake of the prevention and control of soil heavy metal pollution [3].

In recent years, large numbers of studies have been conducted on soil and sediment potentially toxic element pollution and risk assessment, however, studies focused on metal pollution and risk in drinking water source areas are relatively few. The Miyun Reservior is the most important drinking water source for Beijing in China, and thus its soil and aquatic environmental safety are essential to the health of its residents. To our knowledge, some studies have been reported on potentially toxic element pollution levels, remobilization characteristics and source identification [22,23,24]. However, no work has been reported the health risks induced by potentially toxic elements of soil and sediment in the Miyun Reservoir. Herein we present our study assessing the potentially toxic element pollution level, ecological risk, and human health risk of soils and sediments in the Chao River and Baimaguan River, which are located upstream of the Miyun Reservoir. The results of this study will provide useful reference information for potentially toxic element pollution control and risk management in this region.

## 2. Materials and Methods

### 2.1. Study Area

The study area is located in northern Miyun County, Beijing, China, upstream of the Miyun Reservoir. The Miyun Reservoir (40°23′ N, 116°50′ E) was built in 1960 and has a surface area of 188 km^2^, with a catchment area of approximately 15,788 km^2^. It is the largest reservoir in northern China and consists of eastern and western reservoirs, as well as an enclosed independent water body referred to as the inner lake. Two main rivers, the Chao and the Bai, flow into the Miyun Reservoir. Surface runoff roughly mirrors precipitation, with large interannual fluctuations and an uneven distribution throughout the year. The Miyun Reservoir has been used for Beijing’s drinking water storage since 1997. The water quality in Miyun Reservoir directly affects the health and safety of the residents of Beijing.

### 2.2. Sampling and Analyses Methods

**Figure 1:**
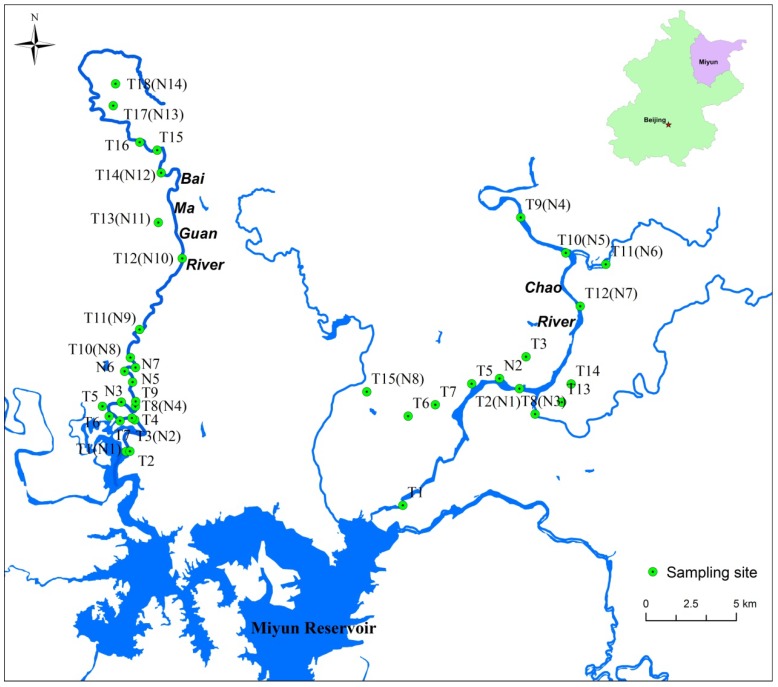
Distribution of sampling sites.

Soil samples were collected using the method described by Pan et al. [25]. Briefly, in July 2016, a total of 33 surface (0–20 cm) soil samples and 18 sediment samples were collected from farmland soil along the Chao River and Baimaguan River (Figure 1). At each sampling site, five subsamples were taken from the same area (approximately 100 m^2^) and thoroughly mixed to form one composite sample. The samples were placed into self-sealing polyethylene bags and transported to the laboratory. The positions of the sampling sites were recorded using a hand-held global positioning system. All soil and sediment samples were placed in polyethylene bags and brought to the laboratory. Then the samples were air-dried for 3 weeks at room temperature in a storage room, passed through a 60-mesh sieve after removing stones, residual roots, and other unwanted materials, and then sealed in brown glass bottles and stored in a refrigerator at −4 °C until subsequent analysis. Samples were analyzed following methods 3050B and 6010C proposed by the United States Environmental Protection Agency [26], respectively. Briefly, a subsample (approximately 10 g) was air-dried, and gently ground using an agate pestle, and passed through a 100-mesh nylon sieve. A total of 1 g sample was placed into a PVC digestion vessel (Anton Parr, Graz, Austria) along with 9 mL of concentrated nitric acid (HNO_3_) and 3 mL of hydrogen peroxide (H_2_O_2_). The vessel was then sealed and heated at 180 °C for 15 min. The concentrations of six potentially toxic elements, namely As, Cd, Cr, Cu, Pb, and Zn in the digestion solution were determined using inductively coupled plasma mass spectrometry (POEMS3; Thermo Electron, Waltham, MA, USA). Quality assurance and control (QA/QC) measures included procedural blanks, duplicate analyses and standard reference materials. Soil standard reference material (GBW07401, GSS-1) obtained from the Center of National Standard Reference Material of China was used for QA/QC. Accepted recoveries ranged from 81.0% to 109%. Statistical analyses were conducted using Microsoft Excel (Microsoft Inc., Redmond, VA, USA) and Origin 9.0 (OriginLab Corporation, Northampton, MA, USA).

### 2.3. Statistical Analyses

The enrichment factor (EF) is considered an effective tool for evaluating the amount of contaminants in an environment. Potentially toxic element pollution levels were assessed using EFs and the geoaccumulation index (Igeo). According to Sutherland [27], EF values are calculated with the following equation:

EFs = C_i_/B_i_/C_B_/B_B_,

where C_i_ and C_B_ is the i-th potentially toxic element concentration (mg/kg) and background value (mg/kg) in the soils, respectively. B_i_ and B_B_ is the reference potentially toxic element concentration (mg/kg) and background value (mg/kg) in the soils. According to Chen et al. [2], in this study, Al was adopted as reference because it is one of the largest components of soil. Six contamination categories are generally recognized on the basis of enrichment factors (Table 1):

Igeo is a geochemical criterion introduced by Müller [28], which can be used to evaluate soil contamination by comparison between current and preindustrial concentrations. Unlike other methods of pollution assessment, Igeo takes the natural diagenetic process into account, which makes its assessments more realistic. Igeo is calculated using the following equation:

Igeo = log2(Cn/1.5 × Bn)

where Cn is the measured concentration of the potentially toxic element in soil (mg/kg), Bn is the geochemical background value of the corresponding potentially toxic element (mg/kg), and the coefficient 1.5 is used due to potential variation in the baseline data [29]. According to Müller [28], Igeo values fall into seven classes. The corresponding relationships between Igeo and the pollution level are listed in Table 2.

### 2.4. Ecological Risk Assessment Method

The method introduced by Hakanson was adopted with the aim of assessing the ecological risks posed by potentially toxic element pollution in the topsoil of the study area. Hakanson [30] developed the following quantitative approach, and this method was used in many other studies to assess ecological risks of potentially toxic elements in soils [31]. The potential ecological risk factor of a given contaminant (E(i)) is defined as:

EI(i) = T(i) × C_i_/C_0_

where Ti is the toxic-response factor for a given substance (i.e., Cd = 30, As = 10, Pb = Cu = 5; Cr = V = 2; Zn = 1), C_i_ represents the potentially toxic element content in the topsoil, and C_0_ is the regional background potentially toxic element content of topsoil. The sum of the individual potential risk factors (E(i)) is the potential ecological risk index (RI), which represents the potential risk in a region. RI can be expressed as:

$$RI=\sum_{i=1}^{n}T(i)$$

### 2.5. Human Health Risk Assessment

(1) Exposure assessment

In general, individuals are exposed to contaminants through three pathways: ingestion, inhalation and dermal contact, which can be determined according to the Exposure Factors Handbook [32]. However, for potentially toxic elements in soil, ingestion and dermal absorption are considered the main exposure pathways [33]. Thus, these two exposure pathways were selected for health risk assessment in this study. The average daily intake (ADI) of chemicals from soils is calculated using the following equations:

$$ADI_{\mathrm{non-dietary}}=\frac{C\times IR_{\mathrm{ing}}\times EF\times ED}{BW\times AT}\times 10^{-6},\;ADI_{\mathrm{dermal}}=\frac{C\times SA\times SAF\times ABS\times EF\times ED}{BW\times AT}\times 10^{-6}$$

where C is the concentration of the potentially toxic elements in soil (mg kg^−1^); IR_ing_ is the ingestion rate (mg day^−1^), 100 mg kg^−1^ for adults and 200 mg kg^−1^ for children [34]; EF is the exposure frequency, 350 days/year [34]; ED is the exposure duration, 30 years for adults and 6 years for children [34]; SA is the exposed area, 5700 cm^2^ for adults and 2800 cm^2^ for children [34]; SAF is the adherence factor, 0.07 mg/cm^2^ for adults and 0.2 mg/cm^2^ for children [34]; ABS is the dermal absorption factor, 0.001 for all elements considered in this study [34]; BW is body weight, 70 kg for adults and 20 kg for children [34]; AT is the averaging time, determined for non-carcinogens as ED × 365 days and for the carcinogens As and Cr as 70 (lifespan) × 365 days [34].

(2) Non-carcinogenic risk assessment

The hazard quotient (HQ) is traditionally used to describe non-carcinogenic risk, which is calculated as the ratio of the average daily dose and the reference dose (RfD) for a given substance. The equation is defined as follows:

$$HQ=\frac{ADI}{RfD},$$

where RfD is the reference dose of the i-th potentically toxic element (mg/kg day^−1^), as listed in Table 3, which is the maximum allowable level of the potentially toxic element that causes no harmful effects on human health. In this study, the RfD_ing_ value in soil for both adults and children are taken into consideration. Because there are no reference doses for evaluating dermal absorption exposure to chemicals, USEPA [34] provides a method to assess dermal risk by multiplying the soil oral reference doses with a gastrointestinal absorption factor. To assess the overall non-carcinogenic effects of multiple chemicals, the sum of HQ values for all chemicals are expressed as HI, following the equation:

$$HI=\sum HQ_{i}=\sum \frac{ADI_{i}}{RfD_{i}}$$

if the HI value is less than 1, the exposed individual is unlikely to experience obvious adverse health effects. On the other hand, if the HI value exceeds 1, there is a chance that non-carcinogenic effects may occur, with a probability that tends to increase as HI increases.

(3) Carcinogenic risk assessment

Carcinogenic risk is defined as the probability of an individual developing any type of cancer throughout their lifetime due to exposure to carcinogenic hazards. The carcinogenic risk for an individual over a lifetime is calculated according to the following equation [35]:

CR=ADI×SF,

where the SF is the carcinogenicity slope factor (per mg/kg^−day^) as also listed in Table 3, if multiple carcinogenic contaminants are present, the cancer risks from all chemicals and routes are summed. Risks between 1.0 × 10^4^ and 1.0 × 10^6^ are generally considered acceptable [36], while those exceeding 1.0 × 10^4^ are considered to represent a lifetime carcinogenic risk to the human body.

## 3. Results

### 3.1. Potentically Toxic Element, Nitrogen, and Phosphorus Concentrations in Soil and Sediment Samples

Table 4 presents the statistical characteristics of potentially toxic elements, total nitrogen, and total phosphorus concentrations in the soil and sediment of the Chao and Baimaguan Rivers, along with background concentrations in the Beijing area. The concentrations of six potentially toxic elements in the soil varied greatly in the study area; chromium was found at the highest concentrations, followed by Zn, Cu, Pb, and As. Concentrations of Cd were considerably lower than those of other metals. The average concentrations of Cd, Pb, Cr, As, Zn, Cu, total nitrogen and total phosphorus were all below the Chinese Environmental Quality standards for potentially toxic elements in soils [37].

However, it should be noted that the concentrations of Cd in the soil were 0.14 mg/kg, which were 2.58 times greater than background values in Beijing. A similar trend was also observed for As and Cu, which both had concentrations 1.09 times greater than their background values. These results were consistent to another study conducted in Chao-Bai River of Miyun Reservoir. According to Han et al. [22], Cd was also the most heavily polluted metal in the soil with the geometric mean of Cd being 2.6 times higher than the background value for Beijing, and the mean concentrations of Zn, As, Hg, and Pb were comparable to the soil background values. In sediment, as listed in Table 4, Cd was the most heavily polluting metal, exceeding 3.40 times its background values, while Pb and As were 1.34 and 1.61 times greater than their background values, respectively, while Cr, Cu, and Zn contents were all lower than background values. Generally speaking, the concentrations of potentially toxic elements in the study area are not high, with the exception of Cd.

### 3.2. Potentially Toxic Element Pollution Levels in the Soil and Sediment

**Figure 2:**
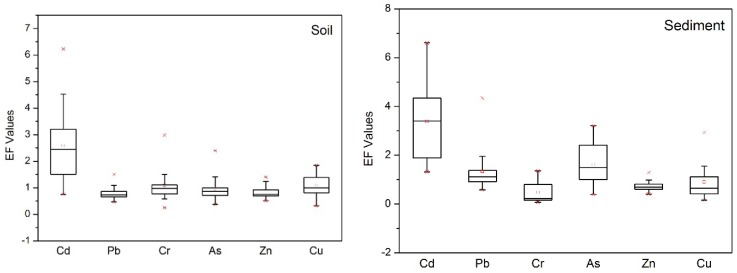
The EF values of potentially toxic elements in soil and sediment (The upper **×** indicates 95th percentile and the lower **×** indicates 5th percentile value, respectively □ indicate median value).

**Figure 3:**
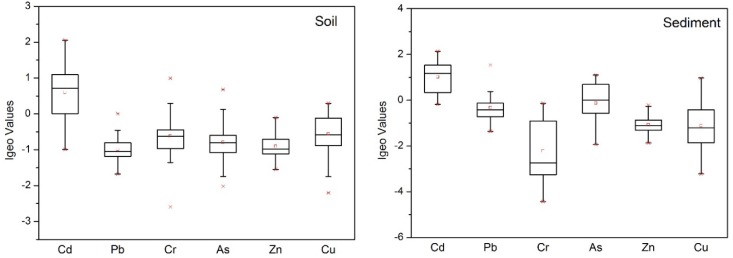
The Igeo values of potentially toxic elements in soil and sediment (The upper **×** indicates 95th percentile and the lower **×** indicates 5th percentile value, respectively □ indicate median value).

The contamination levels of potentially toxic elements were assessed using the EF and Igeo index. The calculated EF and Igeo values for potentially toxic elements in soil and sediment are summarized in Figure 2 and Figure 3. In soil samples, the mean EF values of Cd, Pb, Cr, As, Cu, and Zn were 2.58 (0.75–6.23), 0.77 (0.47–1.51), 1.09 (0.25–2.99), 0.95 (0.37–2.40), 0.83 (0.51–1.40), and 1.09 (0.33–1.84). The EF values of soil Cd in 3.03%, 66.67%, and 18.18% of samples represented significant pollution, moderate pollution, and slight pollution, respectively. Among the other potentially toxic elements, 51.12% of samples indicated slight pollution with Cu, while 9.09% and 33.33% showed moderate and slight pollution with Cr, 3.03% and 21.21% exhibited moderate and slight pollution with As, 21.21% had slight pollution with Zn, and 9.09% showed slight pollution with Pb. The average Igeo values for Cd, Pb, Cr, As, Cu and Zn in the soil were 0.60, −0.99, −0.62, −0.79, −0.91 and −0.56, respectively, which implies that except for Cd presenting as unpolluted to moderately polluted, all potentially toxic elements were present at unpolluted levels. For Cd, 45.5% of samples showed moderate pollution and 3.03% were heavily polluted.

In sediment samples, the mean EF values of Cd, Pb, Cr, As, Cu, and Zn were 3.39 (1.32–6.60), 1.34 (0.58–4.34), 0.47 (0.07–1.36), 1.61 (0.39–3.22), 0.74 (0.41–1.29) and 0.91 (0.16–2.94), respectively, which indicates moderate pollution with Cd, slight pollution with Pb and As, and no pollution with Cr, Cu or Zn. Overall, sediments in the Chao and Baimaguan Rivers showed the same pollution trends as the soil. In particular, 16.67% of samples showed significant Cd pollution, while 55.56% and 27.8% showed moderate and slight pollution, respectively. The average Igeo values for Cd, Pb, Cr, As, Cu, and Zn in the sediments were 1.02, −0.33, −2.22, −0.13, −0.18, and −1.11, respectively, which indicates that except for moderate Cd pollution, all other potentially toxic elements fell into the unpolluted category.

### 3.3. Relationships between Potentially Toxic Elements, Total Phosphorus, and Total Nitrogen

Relationships between the contents of potentially toxic elements, total phosphorus, and total nitrogen were investigated and shown in Table 5. Based on Spearman correlation coefficients, Cd, Pb and As were closely related (*p* < 0.01), as were Cu and Cr (*p* < 0.01). Significant correlations between metals could result from shared pollution sources [25]. As shown in Table 5, the contents of Cd and Pb were very closely related (*p* < 0.01) to that of total nitrogen in both soil and sediment samples. Total nitrogen concentrations in the soil could affect soil pH. Generally speaking, through the action of rainfall and irrigation water, nitrogen that enters the soil is mostly rinsed into the subsoil in the soluble NO_3_^−^, NO_2_^−^, and NH_4_^+^ forms. Under proper conditions, nitrogen can be completely nitrated rapidly. These reactions have a significant impact on soil pH; when NH_4_^+^-N enters the soil, its pH can be significantly reduced in the short term due to nitrification. Young et al [39] found that nitrification of only NH_4_^+^-N could reduce the topsoil pH by 0.2 to 1.4 units. Breteler and Smit [40] found that the pH of rhizosphere solution increased by 1.87 units under pure culture conditions with NO_3_^−^-N. Overall, the impact of nitrogen application on the soil is mainly reflected in the pH. However, more research should be conducted into the relationship between high nitrogen content and potentially toxic element levels in soils and sediments.

### 3.4. Potential Ecological Risk

(1) Soil

**Figure 4:**
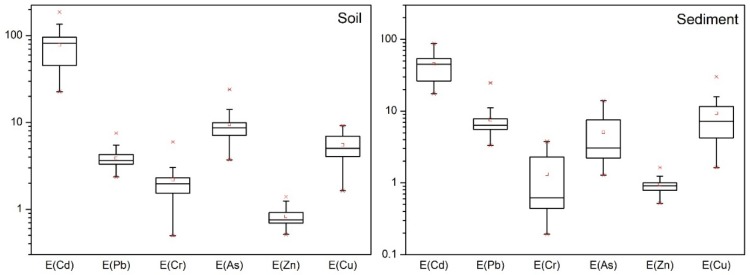
The EI values of the potentially toxic elements in soil and sediment (The upper **×** indicates 95th percentile and the lower **×** indicates 5th percentile value, respectively □ indicate median value).

Figure 4 shows the enrichment index (EI) of potentially toxic elements in soil from the Chao and Baimaguan Rivers. The potential ecological risk posed by the six potentially toxic elements varied significantly. The mean EI values of Cd, Pb, Cr, As, Zn and Cu in Chao River soil were 101.51, 3.84, 2.80, 9.19, 0.73, and 6.43, while those for the Baimaguan River were 57.55, 3.90, 1.65, 9.78, 0.91 and 4.65, respectively. In general, the ecological risks posed by potentially toxic elements in Chao River soil were higher than those in Baimaguan River soil, and the primary element posing the greatest potential ecological risk was Cd. By contrast, the other five potentially toxic elements showed low potential ecological risks. According to Hakanson, the potential ecological risk posed by Cd in Chao and Baimaguan River soils reflected considerable potential ecological risk and moderate potential ecological risk, respectively. It should be noted that 81% of all samples for E(Cd) from the Chao River presented considerable potential ecological risk, while 61% of E(Cd) samples from the Baimaguan River indicated moderate to considerable potential ecological risk.

(2) Sediment

In sediments (Figure 4), the mean EI values of Cd, Pb, Cr, As, Zn, and Cu in the Chao River were 50.94, 5.88, 2.39, 8.54, 0.94, and 11.68, whereas for those in the Baimaguan River were 40.00, 8.98, 0.45, 2.22, 0.95 and 7.44, respectively. Similar to the trend observed in soil, Cd was the element posing the greatest eco-risk. Although the potential eco-risks posed by sediments were lower than those of soil, the EI values of Cd indicated moderate and low potential eco-risk in the Chao River and Baimaguan River, respectively. Similarly to soil, the other five potentially toxic elements showed low potential ecological risk in sediments. Sediment texture, salinity, and organic content are responsible for the mobility and availability of metals in the superficial sediment layer [41]. Generally speaking, sediment organic carbon is often viewed as a major sink or carrier of potentically toxic elements due to its strong capacity form complexes with metallic contaminants. Both the release and bioavailability of contaminants present in sediments can be affected by natural and anthropogenic disturbance events. Potentially toxic elements in mangrove sediment are likely to be released and transported into the water when soil physicochemical properties change, leading to an environmental hazard [42]. In addition, as shown in Figure 4, the sampling sites near iron ores did not present high potentially toxic element concentrations, which indicates that the correlation between mining activity and potentially toxic element concentration in the sediment is negative.

### 3.5. Human Health Risk Posed by Soils

(1) Non-carcinogenic risks

The human health risks posed by potentially toxic elements in Chao River and Baimaguan River soils were also calculated. According to the USEPA classification of carcinogens and non-carcinogens, Cd, Pb, Zn, and Cu were investigated for their non-carcinogenic risks^−^ while Cr and As were assessed for their carcinogenic risks. Table 6 presents the non-carcinogenic risks associated with non-dietary ingestion, dermal contact, and inhalation due to potentially toxic element exposure in soils. For the Chao River, the average values of Cd, Pb, Zn and Cu were 2.86× 10^−4^, 1.96 × 10^−2^, 2.47 × 10^−3^ and 1.36 × 10^−4^ for adults and 1.74 × 10^−3^, 1.36 × 10^−1^, 1.71 × 10^−2^ and 9.48 × 10^−4^ for children, respectively. Meanwhile, the average values of Cd, Pb, Zn and Cu in the Baimaguan River were 1.62 × 10^−4^, 1.87 × 10^−2^, 3.06 × 10^−3^ and 1.04 × 10^−4^ for adults and 9.86 × 10^−4^, 1.29 × 10^−1^, 2.12 × 10^−2^ and 7.21× 10^−4^ for children, respectively. 

Among the potentially toxic elements studied, Pb appeared to pose significantly greater non-carcinogenic risks to urban residents than the other four potentially toxic elements due to its high concentration in soils or low RfD values. Overall, the non-carcinogenic risks of the six potentially toxic elements were all much lower than 1, which suggests that there would be no non-carcinogenic risk in this area. Compared to adults, children have higher HI values for non-carcinogenic risk because children are more susceptible to a given dose of toxin and likely inadvertently ingest significant quantities of soil due to pica and hand- or thumb-sucking behaviors. For example, children sometimes accompany their parents to farmland where they are exposed to a contaminated environment without any protective measures, exposing them to soils containing potentially toxic elements [4]. In addition, the HI values of all potentially toxic elements for non-dietary ingestion by children were hundreds of times greater than those via dermal contact and inhalation, and a similar trend was also observed for adults, which indicates that non-dietary ingestion is the predominant exposure pathway of potentially toxic elements both for adults and children, consistent with the results of previous studies [43,44]

(2) Carcinogenic risk

As shown in Table 6, the carcinogenic risks of Cr and As for adults were 3.21 × 10^−5^ and 7.69 × 10^−6^ in the Chao River, and 1.90 × 10^−5^ and 8.18 × 10^−6^ in the Baimaguan River, respectively, which were lower than those for children (4.28 × 10^−5^ and 1.07 × 10^−5^ in the Chao River, 2.53 × 10^−6^ and 1.14 × 10^−5^ in the Baimaguan River, respectively). The combined carcinogenic risk for children via non-dietary ingestion, dermal contact and inhalation is 1.40 times greater than for adults with respect to average risk values, highlighting that children may suffer more carcinogenic risks in their daily life via unconscious ingestion pathways. According to Fryer et al. [33], risks greater than 1 × 10^−4^ are viewed as unacceptable, whereas risks below 1 × 10^−6^ are not considered to pose significant health effects, and risks lying in the range of 10^−6^–10^−4^ are generally considered tolerable. The majority of the carcinogenic risk lay between 1 × 10^−6^ and 1 × 10^−5^ the cancer risks obtained for adults and children were all within the acceptable range. Therefore, it can be concluded that no serious long-term health impacts to adults are expected due to Cr and As in soils of these rivers. Although the health risks of the six potentially toxic elements in the soil investigated were all within acceptable ranges, Cd requires attention from environmental protection agencies, as it is the most heavily polluted element in both the soil and river sediment.

## 4. Discussion

Compared with the potentially toxic elements concentrations in the soil and sediments of other nationally important water sources, the potentially toxic element concentrations in the upstream of Miyun Reservoir seems lower. For example, the soil Cd, As and Cu in Danjiangkou Reservoir located in central China, were measured in the range of 0.02–1.17, 14.8–48.7, 17.03–52.4 mg kg^−1^, and the average values were 0.27, 24.28, 27.76 mg kg^−1^, respectively [45]. The mean contents of Cu, As and Cd in the Danjiangkou Reservoir were the main pollution elements in arable land and were strongly affected by anthropogenic sources (such as agricultural fertilizers and pesticides) and were higher than other land-use types, showing the same trend to the Miyun Reservoir. According to the study of Han River from Liu et al. [11], the average concentrations of Cd, Cr, Cu, Pb, Ni and Zn in the soils along the Han River were 0.54, 84.6, 37.7, 25.4, 111, and 95.5 mg/kg. Land uses had the most significant effect on the metal concentrations. Another study focused on Poyang Lake in Jiangxi Province, the contents range of Cu, Zn, Pb, Cd, Cr, and As were 37.92–709.28, 70.23–362.64, 54.26–160.63, 0.49–8.79, 51.32–153.63, and 9.72–47.66 mg/kg. Compared to rivers in the other developing countries, the metal contents in soils of the upstream of Miyun Reservoir also presented lower levels. For example, the concentration of Cd, Cr, Cu, Ni, and Pb in soils along Curu River watershed in Brazil were 0.38, 130, 34.6, 88, and 19.8 mg/kg, respectively; As for another research of the Khorat Basin in the northeast Thailand, the average concentration of As, Cd, Cr, Cu, Pb, and Zn were 1.7, 0.34, 64, 20.7, 23.3, and 61mg/kg, respectively. Application of fertilizers is the main reason for Cd, Cu and Zn pollution in the soil. According to research by Zhang [46], Cd reached significant enrichment levels (5 < EF < 20), and approximately 97.14% of soil samples were heavily polluted with Cd in the Yellow River Delta of China. EF values for Cd in all soil samples were greater than 1.5, which means that Cd pollution in the YRD might be seriously affected by anthropogenic inputs. Similar results were found in the study by Zhang [47], wherein average EF values for both Cd and Pb in the Bortala River in Xinjiang indicated extreme pollution, and the average ecological risk indices for Cd and Pb were 73 and 95, respectively, which indicated moderate pollution. High concentrations of Cd and Pb were primarily distributed in sediments from certain land use types, generally farmland and built-up urban areas with high populations and economic activity.

As for the sediment, there are also quantities of studies focused on potentially toxic elements. For example, a study conducted in five reservoirs in Liaoning and Jilin Province, which located in northern China, were detected for the sediment potentially toxic elements [48]. The concentration ranges for Cu, Cd, Pb, Zn and Cr were 26.20–42.74 mg/kg, 1.33–2.43 mg/kg, 38.38–82.40 mg/kg, 101.11–148.06 mg/kg and 71.97–113.59 mg/kg, respectively. The contamination of potentially toxic elements in the sediments is higher than those in Miyun Reservoir, showing a closely relation to the mining industries located in the upstream basins. As for the western region in China, for example, Zhang et al. studied the sediment potentially toxic elements in the Bortala River in Xinjiang Province, the concentration of Cu, Pb, Cd, Zn, As and Cr were 30.09, 31.98, 0.17, 99.19, 9.67, 51.55 mg/kg, Cd, Hg, and Pb showed the similar concentration with those in Miyun Reservoir, and presented the main potential ecological risk factors with contributions of 25.43, 22.23%, and 21.26%. There are also other studies focused on potentially toxic elements pollution in the Miyun Reservoir, for instance, Qiao et al. [49] showed that the average concentrations of sediment Cu, Cd, Pb, Zn, Cr, and As in Chao River, the largest tributaries of the Miyun Reservior, were 69.80, 3.32, 20.49, 94.79, 54.48, 4.62 mg/kg, respectively. Their values are significantly higher than our results, which was attributed to that the samples sites of Qiao et al. [49] were located in the inflow and outflow tributaries of the Miyun Reservoir. However, in another study conducted for sediment core in the Miyun Reservior by Wu et al. [13], the average concentration of Cd, Cr, and Zn are 0.028, 21.74, and 19.28 mg/kg, respectively. While in this research, the sampling sites are in the reservoir area, the water volume of which rises significantly after South-to-North Water Transfer Project, and are less affected by the potentially toxic elements concentration of tributaries. Another study conducted in the Pearl River Estuary of China by Zhang et al. [50] showed that the Igeo values for Cd in all sediments of urban, rural and reclamation-affected rivers were categorized as “heavily to extremely polluted” (4 < Igeo < 5). Dusts and aerosols stemming from human activities, such as industrial and energy production, construction, vehicle exhaust and waste disposal are easily deposited into surrounding river sediments where they cause serious metallic pollution, and were considered the main source of potentially toxic element pollution in Pearl River sediments. Generally speaking, Cd was considered the primary contaminating metal in both soil and sediments samples from the Baimaguan and Chao Rivers. Its concentration was most likely influenced by extrinsic factors, such as human activities, automobile exhaust, and deposition of aerosols [31]. The results described above are in accordance with the survey conducted by Chen et al. [2], who reported that Cd and Hg are the main elements contributing to soil potentially toxic element contamination in China. In particular, increased anthropogenic inputs caused by rapid economic development since the last 1970s, establishment of industrial operations, and rapid urban expansion in China have drastically increased industrial and municipal wastewater discharges. In addition, similar trends in potential ecological risks were also found in other studies. For instance, among the potentially toxic elements assessed, namely As, Cd, Cr, Cu, Hg, Pb, Sb, and Zn, in soils from Wen’an County, Hebei Province in China, the potential ecological risk caused by Cd was greatest, and Cd posed considerable potential eco-risk (E(Cd) 125; [51]). In the soils of other megacities, such as Beijing and Shenzhen, cadmium was also observed at much higher levels than other potentially toxic elements investigated with respect to potential ecological risk.

Overall, the comparison above reflects the variation in potentially toxic element concentrations among different regions, as well as the impact of anthropogenic activities on potentially toxic element enrichment in sediments.

The above results support treating Cd with concern in terms of risk to the ecosystem of Miyun Reservoir. The area of highest eco-risk was at the entrance of Miyun Reservoir, which may be influenced by dust, solid waste, accumulated mill tailings and acid drainage generated by mining, smelting and other activities. As is evident from the survey results, there are several iron ore mines in upstream of Miyun Reservoir, mainly distribute in the Chao River and Baimaguan River catchments, and mining history has been going on for more than 30 years. Since 2005, these iron ore mines has been closed one after another, despite this, due to the chronic deposition of potentially toxic elements in soil, there is still residual contamination in the soil surrounding these iron ore mines. Combined with the effect of soil erosion under rainfall on agricultural land in Miyun Reservior, which preferentially carries away potentially toxic elements and other organic matter. Ultimately, the carried potentially toxic elements import into aquatic environment, which furthermore induced potentially toxic elements accumulation in sediments.

In general, enrichment of Cd is considered to be caused by agricultural activities, including livestock and poultry breeding, as well as pesticide and fertilizer application. It was found that there are some fishponds and large numbers of orchards, and especially for orchards, large amounts of fertilizer are used, which could induce soil potentially toxic element contamination. According to another study in the Chao River in the upstream of Miyun Reservoir, the sources of Cd in the soil were identified by the PCA and cluster analysis methods and the results showed that the factor loading of Zn was very close to that of Cd, and distribution map also display a similar uniformity of these two elements’ distributions. Weathering erosion of the parent rock, mining activity and fertilizer application were the main origins of Cd and Zn [23]. For the sediment Cd, it also has been identified by Wu et al. [13], in the study of which found that due to agricultural land is the major land use type in Miyun Reservoir catchment. Thus, Cd and Zn in sediment were mainly from the phosphate fertilizer-related non-point source pollution. In addition, there are very few resident homes and tourist activities around, suggesting little domestic sewage discharge would be another source. Overall, phosphorous fertilizer and soil erosion are the two main sources for Cd pollution in the soils and sediments of the Miyun Reservoir.

In recent years, the Beijing Government has enacted a series of policies and strategies to control potentially toxic element pollution, such as industrial restructuring, clean energy development and coal consumption controls. Particularly in Miyun District, the most important drinking water source in Beijing, the assessment and enforcement of potentially toxic element pollution is strict. Therefore, the contribution of potentially toxic element pollution from other adjacent places should be paid attention, particularly Cd. For example, Hebei Provinces and Tianjin City, which are areas with more serious potentially toxic element pollution. Atmospheric deposition is an important pathway of soil and sediment contamination [52]. Key pollutants such as potentially toxic elements, which can be related to industrial or transportation emissions, will either accumulate directly on ground surfaces or initially accumulate in the atmosphere. Over time, these atmospheric pollutants will also be deposited on ground surfaces for eventual transport by rivers and groundwater. Eventually, potentially toxic elements in the water are absorbed by organic matter in the sediment.

Potentially toxic element pollution control is arduous, and large-scale control must be carried out by governments. China has promoted coordinated management in Beijing-Tianjin-Hebei, namely establishing a collaborative governance mechanism and system. However, achieving successful treatment may take several decades. In addition, it should be noted that a number of other emerging metal contaminants (e.g., platinum, rhodium, and palladium) should also be considered in future environmental studies, as they are now poorly understood in terms of potential ecological and human health risks. Most previous studies on these pollutants have focused on detection methods and on their chemical forms in the environment.

It should be noticed that although the risk assessment model used in this study are useful and powerful tools to distinguish the toxic chemicals and various exposure pathways in the environment, however, the risk assessment of heavy metals in this study remained some uncertainties, which are inherent in quantitative risk assessment. First, the bioavailable or bioaccessible concentration of heavy metals is considered more reliable and accurate in ecological and human risk assessments [53] which suggests that the human risk are assessed based on the total concentration of heavy metals; In addition, organic matter can absorb Cu, Pb, and Cd through its groups such as -RCOOH and -ROH, which made these elements no more be available, leading to the assessed risks in this study may be overestimated [54]. In future work some models also can be considered to be used to avoid these uncertainties, such as study form Di Bonito [55]. However, despite a lack of a completely accurate risk assessment, this study scored the health effects based on a well-defined investigation on oral ingestion and dermal contact exposure pathways and various heavy metals leading to potential ecological and human health risks in a typical county.

## 5. Conclusions

Quantification and assessment of heavy metal pollution and risk in the upstream of Miyun Reservoir is of great importance in ensuring a reliable drinking water supply for the inhabitants of Beijing. The average concentrations of the six potentially toxic elements were all lower than the soil environmental quality standards in China. Cadmium was the most serious pollutant in both soil and sediment, and the potential ecological risk posed by Cd in Chao and Baimaguan River soils were considerable and moderate, respectively. Overall, the potentially toxic element contents level in the upstream of the Miyun Reservoir were lower than most of the other rivers and lakes in China and other developing countries. The historical iron ore mining and agricultural activity were considered the primary sources of potentially toxic element pollution in soils and sediments in the study area. There are no serious non-carcinogenic or carcinogenic risks to either adults or children at this time, as the observed values were lower than safety threshold levels. Overall, the priority for control of Cd element should be concerned with its threat to the ecosystem in the Miyun Reservoir. For the future work in Miyun Reservoir, the fractionation, speciation and transformation mechanism of Cd in soil-water system should be considered to be studied by some models i.e., WHAM and ECOSAT. Furthermore, a number of other emerging metal contaminants (e.g., platinum, rhodium, and palladium) should be also considered in future environmental studies since nowadays they are very limited.

## Figures and Tables

**Table 1:** Contamination categories on the basis of enrichment factors.

EF Values	EF ≤ 1	1 < EF < 2	2 ≤ EF < 5	5 ≤ EF < 20	20 ≤ EF < 40	EF ≥ 40
Categories	no	slight	moderate	significant	strong	extremely strong

**Table 2:** Contamination categories on the basis of Igeo values.

Igeo Values	Igeo ≤ 0	0 ≤ Igeo ≤ 1	1 ≤ Igeo ≤ 2	2 ≤ Igeo ≤ 3	3 ≤ Igeo ≤ 4	4 ≤ Igeo ≤ 5	Igeo ≥ 5
Categories	unpolluted	unpolluted to moderately	moderately	moderately to heavily	heavily	heavily to extremely	extremely

**Table 3:** Summary of reference dose (RfD) and cancer slope factor (SF) of metals and the metalloid As through oral, dermal and inhalation pathway.

Elements	GIABS ^a^	Oral RFD ^b,c^ (mg·kg^−1^·day^−1^)	Dermal RFD ^b,c^ (mg·kg^−1^·day^−1^)	Inhalation RFD (mg·kg^−1^·day^−1^)	Oral SF (mg·kg^−1^·day^−1^)	Dermal SF ^b,c^ (mg·kg^−1^·day^−1^)	Inhalation SF ^b,c^ (mg·kg^−1^·day^−1^)
Pb	1	1.40 × 10^−3^	5.24 × 10^−4^	5.71 × 10^−5^	NA	NA	NA
Cd	0.25	3.00 × 10^−3^	3.00 × 10^−3^	5.71 × 10^−5^	5.01 × 10^−1^	2.00 × 10^1^	NA
Cr	0.25	NA	NA	NA	NA	NA	4.20 × 10^1^
Cu	0.04	2.00 × 10^−2^	5.40 × 10^−3^	NA	1.70 × 10^0^	4.25 × 10^1^	NA
Zn	1	4.00 × 10^−2^	1.20 × 10^−2^	NA	NA	NA	NA
Ni	1	NA	NA	3.00 × 10^−1^	NA	NA	8.40 × 10^1^
As	1	NA	NA	NA	1.50 × 10^0^	3.66 × 10^0^	1.51 × 10^1^
Hg	0.07	3.00 × 10^−4^	2.13 × 10^−5^	8.57 × 10^−5^	NA	NA	NA

^a^ United States Environmental Protection Agency (USEPA) [26]; ^b^ United States Environmental Protection Agency (USEPA) [38]; ^c^ Ferreira-Baptista L and Miguel E [38].

**Table 4:** The statistical characteristics of potentially toxic element, total nitrogen, and total phosphorus concentrations in the soil and sediment (mg/kg).

Metals	Soil					Sediment					BK
	N	Minimum	Maximum	Mean	SD	N	Minimum	Maximum	Mean	SD	
Cd	33	0.04	0.33	0.14	0.06	18	0.07	0.35	0.18	0.08	0.053
Pb	33	11.60	37.19	19.14	4.95	18	14.42	107.25	33.01	20.46	24.7
Cr	33	16.59	199.25	72.44	37.42	18	4.63	91.01	31.53	26.50	66.7
As	33	3.48	22.55	8.94	4.25	18	3.69	30.24	15.14	8.07	9.4
Cu	33	49.82	135.72	80.46	22.28	18	39.79	125.53	72.34	23.17	97.2
Zn	33	7.52	42.54	25.21	9.02	18	3.70	67.96	21.01	16.96	23.1
TN	33	437.00	1620.00	850.39	271.36	17	119.00	5620.00	1741.53	2024.35	-
TP	33	218.00	3040.00	1165.55	551.50	17	334.00	4380.00	1527.82	994.13	-

TN: Total Nitrogen, TP: Total phosphorus.

**Table 5:** Correlation coefficients of potentially toxic element, total phosphorus and total nitrogen concentrations.

	Metals	Cd	Pb	Cr	As	Zn	Cu	TP	TN
**Soil**	Cd	1.000	0.535 *	−0.185	0.583 *	0.324	0.038	0.140	0.621 *
	Pb		1.000	−0.504	0.495	0.234	−0.079	0.148	0.727 **
	Cr			1.000	−0.175	0.343	0.468	0.189	−0.057
	As				1.000	0.286	0.243	−0.018	0.311
	Zn					1.000	0.836 **	0.146	0.411
	Cu						1.000	−0.118	0.221
	TN							1.000	0.129
	TP								1.000
**Sediment**	Cd	1.000	0.931 **	0.083	0.411	0.011	0.233	0.767 **	0.028
	Pb		1.000	0.036	0.323	−0.009	0.205	0.767 **	0.106
	Cr			1.000	0.009	0.375	0.536 *	−0.112	−0.020
	As				1.000	−0.046	−0.011	−0.038	−0.094
	Zn					1.000	0.552 *	0.154	0.482 *
	Cu						1.000	0.209	0.319
	TN							1.000	0.337

* indicate significant correlation; ** indicate highly significant correlation.

**Table 6:** The non-carcinogenic risk of Cd, Pb, Zn, Cu and cancer risk of Cr, As in Chao River and Baimaguan River soil.

Metal	Chao River		Baimaguan River	
	Adults	Children	Adults	Children
Cd	2.86 × 10^−4^	1.74 × 10^−3^	1.62 × 10^−4^	9.86 × 10^−4^
Pb	1.96 × 10^−2^	1.36 × 10^−1^	1.87 × 10^−2^	1.29 × 10^−1^
Zn	2.47 × 10^−3^	1.71 × 10^−2^	3.06 × 10^−3^	2.12 × 10^−2^
Cr	3.21 × 10^−5^	4.28 × 10^−5^	1.90 × 10^−5^	2.53 × 10^−5^
As	7.69 × 10^−6^	1.07 × 10^−5^	8.18 × 10^−6^	1.14 × 10^−5^
Cu	1.36 × 10^−4^	9.48 × 10^−4^	1.04 × 10^−4^	7.21 × 10^−4^
